# Measurement of Quality-of-Life Outcomes in Pediatric and Young Adult Patients Treated for Eosinophilic Esophagitis

**DOI:** 10.7759/cureus.21675

**Published:** 2022-01-27

**Authors:** Charles B Chen, Jessica Barry, Praveen K Conjeevaram Selvakumar, Sandra Hong, Lori Mahajan, Sarah Worley, Sophia A Patel

**Affiliations:** 1 Pediatric Gastroenterology, University of Missouri, Columbia, USA; 2 Pediatric Gastroenterology, Hepatology, and Nutrition, Cleveland Clinic Foundation, Cleveland, USA; 3 Pediatric Gastroenterology, Bon Secours Hospital, Richmond, USA; 4 Allergy and Immunology, Cleveland Clinic Foundation, Cleveland, USA; 5 Quantitative Health Sciences, Cleveland Clinic Foundation, Cleveland, USA

**Keywords:** psychosocial functioning, pediatrics, quality of life, atopic disorders, eosinophilic esophagitis

## Abstract

Eosinophilic esophagitis is a chronic, immune-mediated esophageal condition that may lead to impairment of quality of life in pediatric and young adult patients. We performed a prospective, cross-sectional study on 40 patients between the ages of 2-21 years with an established diagnosis of eosinophilic esophagitis. The study evaluated physical, emotional, social, and school functioning in patients undergoing treatment with proton pump inhibitors, dietary elimination, or swallowed corticosteroids. There were no statistically significant differences in total or domain-specific quality of life scores between proton pump inhibitors, dietary elimination, and swallowed corticosteroid therapy. Overall, total and domain-specific quality of life were well-preserved in patients with eosinophilic esophagitis, with the highest scores reported in social functioning. There were also no statistically significant associations between clinical, endoscopic, and histologic features and quality-of-life measures.

## Introduction

Eosinophilic esophagitis (EoE) is a chronic, immune-mediated disease of the esophagus, which has been steadily increasing in prevalence since it was first described more than three decades ago [1]. A meta-analysis by Arias et al. reported a prevalence of 11.9 per 100,000 children in studies before 2008 and a prevalence of 30.9 per 100,000 children in studies after 2008 [2]. While there have been many studies on the pathogenesis and management of EoE, far fewer studies have investigated the quality of life of affected children and young adults. Pediatric patients with EoE often report poor health-related quality of life, and the disease burden may extend into multiple domains, including physical, emotional, social, and school functioning. Multiple factors contribute to this, including the severity of the symptoms, complications of the disease, need for frequent endoscopic evaluation, and the burden of long-term therapy [3-4].

A number of studies in recent years have evaluated the impact of disease, treatment, and economic burden on the quality of life in pediatric patients [3,5-6]. However, there is very little research comparing physical and psychosocial outcomes between different types of therapies. This study evaluated the quality of life outcomes for patients with EoE undergoing treatment with proton pump inhibitors (PPI), dietary elimination, or swallowed corticosteroids. The secondary aim of the study was to assess associations between clinical, endoscopic, and histologic features with specific quality-of-life measures.

## Materials and methods

Measures

We performed a prospective, cross-sectional study on patients between the ages of two and 21 years with an existing diagnosis of EoE who were followed by the pediatric gastroenterology clinic at Cleveland Clinic Children’s Hospital. The diagnosis of EoE was established by the presence of greater than 15 eosinophils per high-power field (hpf) on histology. Patients were required to be on a PPI, dietary elimination, or swallowed corticosteroids at the time of enrollment. Patients who were treated with multiple medications or dietary elimination were required to be on their primary therapy for at least four months. The primary therapy was defined as either the current treatment modality (if the patient received only one therapy) or the most recently implemented therapy (if the patient received multiple therapies). It was felt that four months would provide an adequate buffer period that would allow the quality-of-life scores to reflect the effects of the primary therapy. Patients on dietary elimination as their primary therapy were also required to be on a stable diet for at least four weeks at the time of enrollment. Exclusion criteria included patients with a history of congenital or acquired structural anomalies of the esophagus, prior esophageal surgery, or primary esophageal disorder. Additionally, patients with other significant, chronic conditions were excluded.

The Pediatric Quality of Life Inventory 4.0 (PedsQL 4.0) is a questionnaire that has been used to evaluate disease-specific health-related quality of life in pediatric patients with EoE [7]. The questionnaire evaluates physical, emotional, social, and school functioning and includes reports for both children and parents. Scores range from 0 to 100, with higher scores indicating better quality of life. Patients were administered the questionnaire either during an in-person visit or asked the questions verbally during a telephone encounter. Patients who were five years of age or older filled out the questionnaire themselves while parents filled out the questionnaire for patients under five years of age. This cutoff age was established based on a previous study where it was felt that five years of age was sufficient for the independent completion of the questionnaire [5].

Clinical data, including patient, endoscopic, and histologic data, were collected for analysis. Endoscopic and histologic data were collected from both the initial endoscopy and the most recent endoscopy prior to enrollment in the study. Data were also collected on the presence of atopic disorders, including asthma, eczema, allergic rhinitis, and food allergies.

Statistical analysis

PedsQL total scores and subscores were computed according to scoring manuals. Data were described using medians and quartiles for continuous variables and counts and percentages for categorical variables. Treatment groups were compared on quality-of-life measures using non-parametric Kruskal-Wallis tests. The associations between clinical, endoscopic, and histologic factors and quality-of-life measures were assessed using non-parametric Spearman rank correlation coefficients for continuous and ordinal characteristics and Kruskal-Wallis tests for categorical characteristics. All tests were two-tailed and performed at a significance level of 0.05.

## Results

Demographic characteristics

A total of 40 patients were enrolled in the study. The patient characteristics and initial and most recent endoscopic and histologic findings are presented in Table 1 and Table 2. The majority of patients were male (63%) and Caucasian (90%). The median age at diagnosis was 9.7 years and the median time since diagnosis was three years. The most common primary therapy was swallowed corticosteroids (45%), followed by dietary elimination (35%) and PPI (20%). The median length of time on the primary therapy was 1.8 years. Twenty-five percent (25%) of the patients had symptoms for more than one year and 33% of patients had symptoms for less than one year at the time of initial diagnosis. The most common symptoms were dysphagia (50%) and vomiting (38%). Allergic rhinitis (73%) was the most common atopic condition, followed by food allergies (58%), asthma (55%), and eczema (48%). Food allergies were more common in the swallowed corticosteroid group as compared to the PPI group (p=0.015). However, the other atopic conditions were not significantly different between the treatment groups.

**Table 1:** Patient characteristics N: number of patients providing responses Values presented as median (minimum, maximum)

Characteristic		N
Age at diagnosis, years	14.6 (3, 21)	40
Time since diagnosis, years	3 (0, 12)	40
Ethnicity, %		39
Caucasian	90	
African-American	8	
Gender, %		40
Male	63	
Female	37	
Duration of symptoms at the time of diagnosis, %		40
Less than one year	33	
Greater than one year	25	
Unknown	42	
Initial presenting symptoms, %		40
Dysphagia	50	
Food impaction	18	
Abdominal pain	33	
Regurgitation/heartburn	25	
Vomiting	38	
Poor growth/failure to thrive/weight loss	5	
Atopic conditions, %		40
Food allergies	58	
Asthma	55	
Eczema	48	
Allergic rhinitis	73	
None	5	
Primary therapy, %		40
Swallowed corticosteroids	45	
Dietary elimination	35	
Proton pump inhibitor	20	
Duration of primary therapy, years	1.8 (0.3, 8.9)	

**Table 2:** Endoscopic and histologic findings N: number of patients providing responses Values presented as median (minimum, maximum)

Characteristic		N
Initial endoscopy, %		40
Edema	18	
Furrows	60	
Rings	15	
Exudates/Plaques	55	
Normal endoscopy	10	
Other	23	
Most recent endoscopy, %		40
Edema	35	
Furrows	35	
Rings	15	
Exudates/Plaques	33	
Normal endoscopy	30	
Other	8	
Eosinophil count/hpf (initial endoscopy), median		
Proximal esophagus	28	22
Mid esophagus	25	20
Distal esophagus	50	37
Eosinophil count/hpf (most recent endoscopy), median		
Proximal esophagus	1	31
Mid esophagus	0	13
Distal esophagus	11	34
History of esophageal stricture, %	13	40
History of esophageal dilation, %	10	40

The most common endoscopic findings were furrows and exudates on the initial endoscopy and furrows and edema on the most recent endoscopy. Ten percent of the initial endoscopies and 30% of the most recent endoscopies were visually normal. Median eosinophil counts ranged from 25 to 50 eosinophils per hpf on the initial endoscopy and 0 to 11 eosinophils per hpf on the most recent endoscopy. Thirteen percent of patients had a history of esophageal stricture, and 10% required an esophageal dilation.

Quality-of-life outcomes

Quality-of-life outcomes are reported in Tables 3-5. Of the 40 patients, four questionnaires were completed by the parent while the remaining 36 were completed by the patient. PedsQL scores ranged from 67 to 98 in the parent-report questionnaire and 80 to 95 in the child-report questionnaire. The median cumulative quality of life score was 83 for the parent-report questionnaire and 84 for the child-report questionnaire. Overall, the social function scores were highest with median scores of 98 and 95 on the parent and child-report questionnaires, respectively. However, school functioning scores were the lowest on both parent and child-report questionnaires with scores of 67 and 80, respectively. For the parent-report questionnaire, one patient did not attend school and scores were only available for the other three patients. There was no statistically significant difference in total or domain-specific quality of life scores between PPI, dietary elimination, and swallowed corticosteroid therapy.

**Table 3:** Cumulative PedsQL child and parent scores N: number of patients providing responses; PedsQL: Pediatric Quality of Life Inventory Values presented as median (minimum, maximum)

	PedsQL score	N
Child Report		
Physical function	91 (44, 100)	36
Emotional function	90 (45, 100)	36
Social function	95 (65, 100)	36
School function	80 (40, 100)	36
Psychosocial function	85 (58, 100)	36
Total score	84 (58, 99)	36
Parent Report		
Physical function	86 (75, 97)	4
Emotional function	68 (45, 85)	4
Social function	98 (80, 100)	4
School function	67 (33, 83)	4
Psychosocial function	81 (56, 90)	4
Total score	83 (68, 88)	4

**Table 4:** PedsQL scores in eosinophilic esophagitis therapies N: number of patients providing responses; PedsQL: Pediatric Quality of Life Inventory Values presented as median (Q1, Q3)

	N	Proton pump inhibitor	Swallowed corticosteroids	Dietary elimination	p-value
Parent Report					
Physical function score	4	91 (84, 97)	75 (75, 75)	88 (88, 88)	0.41
Emotional function score	4	75 (65, 85)	70 (70, 70)	45 (45, 45)	0.41
Social function score	4	98 (95, 100)	100 (100, 100)	80 (80, 80)	0.32
School function score	3	75 (67, 83)	---	33 (33, 33)	0.22
Psychosocial function score	4	84 (77, 90)	85 (85, 85)	56 (56, 56)	0.41
PedsQL total score	4	86 (85, 88)	81 (81, 81)	68 (68, 68)	0.26
Child Report					
Physical function score	36	97 (72, 97)	91 (81, 97)	91 (88, 100)	0.95
Emotional function score	36	85 (70, 95)	85 (75, 95)	95 (65, 100)	0.90
Social function score	36	98 (90, 100)	95 (85, 100)	95 (85, 100)	0.67
School function score	36	85 (65, 95)	75 (65, 95)	80 (80, 95)	0.81
Psychosocial function score	36	84 (77, 93)	82 (75, 97)	93 (73, 95)	0.99
PedsQL total score	36	89 (75, 96)	84 (78, 97)	91 (79, 97)	0.98

**Table 5:** Correlation between PedsQL scores with age and time since diagnosis N: number of patients providing responses; PedsQL: Pediatric Quality of Life Inventory

		N	Spearman correlation coefficient	95% CI	p-value
Age (years)					
	Physical function score	36	0.20	(-0.14, 0.54)	0.24
	Psychosocial function score	36	0.09	(-0.26, 0.44)	0.60
	PedsQL total score	36	0.12	(-0.23, 0.46)	0.50
Age at diagnosis (years)					
	Physical function score	36	0.16	(-0.19, 0.50)	0.36
	Psychosocial function score	36	0.18	(-0.16, 0.52)	0.30
	PedsQL total score	36	0.19	(-0.15, 0.53)	0.27
Years since diagnosis					
	Physical function score	36	-0.01	(-0.35, 0.34)	0.97
	Psychosocial function score	36	-0.07	(-0.42, 0.28)	0.68
	PedsQL total score	36	-0.08	(-0.43, 0.27)	0.65

There was no correlation between the total quality of life scores and age, age at diagnosis, and time since diagnosis (p=0.5, p=0.27, p=0.65, respectively). When evaluating specific domains, no statistically significant correlation was found between physical and psychosocial function scores with age and time since diagnosis. Neither endoscopic features nor eosinophil counts on the initial and most recent endoscopies were significantly different between the three treatment groups as shown in Table 6. Additionally, there was no difference in the prevalence of esophageal strictures (p=0.83) or the need for esophageal dilation (p=0.24) between the treatment groups. In summary, no statistically significant associations were found between clinical, endoscopic, and histologic features with specific quality-of-life measures.

**Table 6:** Atopic, endoscopic, and histologic features in eosinophilic esophagitis therapies *: Significantly different from proton pump inhibitor **: Significantly different from swallowed corticosteroids N: number of patients providing responses Values presented as median (Q1, Q3)

	N			Proton pump inhibitor	Swallowed corticosteroids	Dietary elimination	p-value
Atopic condition, %							
Food allergies		40		13^**^	72^*^	64	0.015
Asthma		40		38	61	57	0.65
Eczema		40		38	39	64	0.35
Allergic rhinitis		40		50	72	86	0.19
None		40		13	6	0	0.48
Endoscopic features (initial endoscopy), %							
Edema		38		25	11	25	0.56
Furrows		38		75	61	58	0.82
Rings		38		13	17	17	0.99
Exudates/Plaques		38		63	56	58	0.99
Normal endoscopy		38		25	6	8	0.31
Other		38		25	28	17	0.88
Histology (initial endoscopy), median eos/hpf							
Proximal esophagus		22		24 (23, 50)	25 (16, 71)	50 (10, 100)	0.71
Mid esophagus		20		22 (8, 34)	21 (15, 50)	38 (25, 60)	0.41
Distal esophagus		37		34 (27, 44)	46 (24, 80)	65 (50, 100)	0.068
Endoscopic features (most recent endoscopy), %							
Edema		35		33	40	43	0.99
Furrows		35		50	47	29	0.59
Rings		35		17	27	7	0.44
Exudates/Plaques		35		67	33	29	0.30
Normal endoscopy		35		33	27	43	0.80
Other		35		0	13	7	0.99
Histology (most recent endoscopy), median eos/hpf							
Proximal esophagus		31		16 (1, 30)	1 (0, 31)	0 (0, 17)	0.38
Mid esophagus		13		---	0 (0, 30)	8 (0, 25)	0.71
Distal esophagus		34		17 (5, 30)	15 (0, 35)	7 (0, 25)	0.68
History of stricture, %		40		13	17	7	0.83
History of esophageal dilation, %		40		13	17	0	0.24
Duration of primary therapy, months		40		16 (5, 46)	30 (18, 68)	18 (13, 41)	0.33

## Discussion

The impact of EoE in pediatric patients is especially important as poor quality of life may lead to numerous long-term clinical, social, and psychological effects. While a number of studies have assessed the impact of various therapies on quality of life, few have focused on pediatric and young adult patients alone. Flood et al. used caregiver and child questionnaires to evaluate the impact of eosinophilic esophagitis on patients between the ages of two and 17 years [8]. Symptoms of the disease along with treatment were found to have a significant impact on school functioning and participation in social activities. Eighty-one percent of children between the ages of eight and 17 years reported emotional impact from EoE. A review of 64 patients by Harris et al. reported that 69% of children with EoE experienced psychosocial dysfunction with problems ranging from anxiety to sleep difficulty [9]. Klinnert et al. found that a higher number of symptoms at baseline were associated with poorer health-related quality of life and that subsequent therapy lead to improvement in quality of life with decreased symptom burden [6]. Another study by Klinnert et al. of 109 patients with EoE revealed improvement in parent-report quality of life of the affected children and reduction in overall number and severity of clinical symptoms after therapy for one year [5].

In evaluating specific therapies, dietary elimination therapy has been shown to negatively impact the quality of life especially with regards to the necessity of restricting specific foods. Menard-Katcher et al. found that patients with EoE on restricted diets had lower dietary quality-of-life scores compared to those on unrestricted diets as assessed by the Patient Assessment of Upper Gastrointestinal Disorders - Quality of Life questionnaire [10]. This may have a significant impact on a child’s social and school functioning, especially in engaging with peers who do not have any food restrictions. Patients with more refractory disease on dietary elimination likely face even more challenges, including the need for multiple endoscopies or even placement of a nasogastric tube for the administration of elemental formula. Data on the impact of swallowed corticosteroids are limited. Kruszewski et al. compared the quality of life of pediatric patients with EoE at baseline and after six to eight weeks of treatment and found that children treated with swallowed fluticasone showed improvement in PedsQL EoE total symptom scores [11].

Currently, little is known about the impact of PPI on the quality of life in the treatment of EoE. As PPIs are increasingly utilized as initial therapy for EoE, we hypothesized that patients requiring swallowed corticosteroids or dietary elimination may have disease that is more difficult to manage and subsequently have a worse quality of life. However, this current study showed that, in fact, treatment with dietary elimination or swallowed corticosteroids did not result in a statistically significant worse quality of life compared to PPI therapy. This suggests that the need for additional or multiple long-term therapies may not necessarily affect a person’s overall functioning significantly. It is important to note that in our study, patients were treated with swallowed corticosteroids or dietary elimination for at least four months. However, some of the patients had remained on these therapies for years. Patients who had only been on these therapies for shorter periods of time may have had significantly different quality-of-life scores. Long-term therapy may allow children and adolescents to develop various coping mechanisms to adjust to the burden of these treatments. Conversely, the burden of long-term therapy may impair functioning in certain domains that would not be noted in those treated for much shorter periods of time.

The literature on social and school functioning in patients with EoE is limited. Most studies have evaluated overall social and school functioning rather than specific aspects of those domains. Jose et al. found that parents of pediatric patients with EoE reported more school avoidance compared to patients without EoE [12]. Therapy, especially dietary elimination, has been shown to lead to increased worry about embarrassment in social situations [13]. This study showed that despite the burdens of disease, social functioning remained relatively well-preserved. On the other hand, school functioning was poorer than other quality-of-life domains on both parent and child questionnaires. While the PedsQL questionnaire provides a comprehensive assessment of overall functioning, it does not provide very detailed assessments of specific domains of quality of life. For example, the evaluation of school functioning only includes specific questions regarding school attendance and performance of school-related activities. The questions on social functioning only assess the patient’s ability to function at the level of their peers as well as attitudes between the patient and their peers. Therefore, the domain-specific scores obtained in this study are likely only reflective of those particular aspects of social and school functioning.

There is also currently a lack of data comparing the domain-specific quality-of-life measures across various common pediatric gastrointestinal disorders. Varni et al. found that patients with organic conditions reported lower quality-of-life scores compared to those with functional gastrointestinal disorders [14]. Klinnert et al. reported that quality-of-life scores for EoE were similar to those in other patients with gastrointestinal diseases but higher than those seen in patients with eosinophilic gastrointestinal disorders (EGID) [6]. It was thought that the more limited gastrointestinal involvement in EoE compared to other EGIDs likely contributed to higher overall functioning in EoE. It is interesting that poorer school function has been reported in multiple studies on EGIDs. Guardians of children with EGID reported worse emotional and school functioning compared to those of children with other chronic illnesses [15]. Patients with EGIDs averaged 20 more missed school days per year compared to healthy patients [16].

This study presents several questions about the quality of life, which should be further investigated. A number of studies have assessed the impact of EoE on specific aspects of physical and psychological functioning such as dysphagia, pain, anxiety, and depression [6,9,13]. However, it would be beneficial to investigate other aspects of school and social functioning such as perceptions of support from peers and teachers and adequacy of provisions for the patient at school. One factor in school functioning may be that some schools do not provide adequate accommodations for patients such as allowing additional time to finish meals. Another area of future research is to investigate the concordance between family and patient perceptions of disease impact. Our study did not have matched child and parent questionnaires for each individual patient, however, such a study may help determine if caregivers and patients share similar views on physical, psychosocial, emotional, and school functioning.

There were several limitations in this study. Given the cross-sectional nature of the study, the duration of therapy varied significantly between patients. The quality of life of a child who had been treated for years may be quite different from that of a child who had only been treated for four months. Additionally, while we put patients in treatment groups based on their most recent therapy, it is difficult to determine how much of an impact previous therapies had on their overall quality of life. Several patients had been on all three therapies at different times in their lives or were even taking it concurrently. While the “non-primary” therapies likely did not control their EoE disease activity, it is unclear how significantly these therapies affected each individual’s physical and social functioning. This further supports the argument that symptom burden is not associated with histologic findings. Finally, there was also variation within each type of therapy. Some patients on dietary elimination only eliminated one food group while others eliminated multiple food groups. Patients in the latter group may have experienced increased challenges with adherence. Similarly, for the swallowed corticosteroid group, dosages of the medications varied, and some patients were treated with fluticasone while others received budesonide.

It is worth mentioning that a significant portion of this study was performed during the coronavirus disease 2019 (COVID-19) pandemic, which may have also affected the quality of life of the study participants. In particular, perceptions about school and social functioning may have been altered, as many of the children only attended virtual classes during this time. It is unclear how patients felt about missing school at a time when some of them were isolated from their peers. Additionally, the ability to keep up with their studies or pay attention to their teachers could have been a major contributor to their school functioning. Thus, while school functioning scores were the lowest, the results may be quite different in the absence of a pandemic.

## Conclusions

In summary, this study showed that patients who receive more extensive therapy often do not have worse quality of life. Despite the disease burden and the challenges of managing their condition, patients typically continue to maintain good quality of life, especially in social functioning. Physical and emotional functioning are also relatively well-preserved. In comparison to the other quality-of-life domains, school functioning is more negatively affected. The factors that contribute to these findings are unclear and deserve further investigation. Additional resources to help students with EoE become more successful in the classroom and avoid missing school may help raise school functioning scores.
